# Long Non-coding RNAs in the Cytoplasm

**DOI:** 10.1016/j.gpb.2016.03.005

**Published:** 2016-05-06

**Authors:** Farooq Rashid, Abdullah Shah, Ge Shan

**Affiliations:** CAS Key Laboratory of Innate Immunity and Chronic Disease, CAS Center for Excellence in Molecular Cell Science, School of Life Sciences, University of Science and Technology of China, Hefei 230027, China

**Keywords:** lncRNA, mRNA stability, mRNA translation, ceRNA, MicroRNA

## Abstract

An enormous amount of long non-coding RNAs (**lncRNAs**) transcribed from eukaryotic genome are important regulators in different aspects of cellular events. Cytoplasm is the residence and the site of action for many **lncRNAs**. The cytoplasmic **lncRNAs** play indispensable roles with multiple molecular mechanisms in animal and human cells. In this review, we mainly talk about functions and the underlying mechanisms of **lncRNAs** in the cytoplasm. We highlight relatively well-studied examples of cytoplasmic **lncRNAs** for their roles in modulating **mRNA stability**, regulating **mRNA translation**, serving as competing endogenous RNAs, functioning as precursors of **microRNAs**, and mediating protein modifications. We also elaborate the perspectives of cytoplasmic **lncRNA** studies.

## Introduction

Mammalian genome is pervasively transcribed into many different complex families of RNA. However, less than 2% of mammalian genome is transcribed into mRNA to encode proteins, whereas a major portion of the genome is transcribed into interweaved and overlapping transcripts that include thousands of non-coding RNA (ncRNA) transcripts [1], [2]. ncRNAs more than 200 nucleotides in length are called long ncRNAs (lncRNAs), which are often transcribed by RNA polymerase II [3], [4]. These lncRNAs are usually devoid of open reading frames (ORFs), with or without the 3′ polyadenylation [5], [6], [7], [8]. Interestingly, expression of lncRNA is more tissue-specific than that of mRNA [9].

In the last several years, a large number of nuclear lncRNAs have been discovered. These lncRNAs play diverse roles in the nucleus through various mechanisms [10]. For example, nuclear lncRNAs control the epigenetic state of particular genes [11], participate in transcriptional regulation [12], get involved in alternative splicing and constitute subnuclear compartments [13], [14].

Although for most if not all of the lncRNAs, nucleus is the place of biogenesis and processing, cytoplasm is the final residence and site of action for some lncRNAs. Biogenesis of lncRNAs is quite complicated and share many features of protein-coding RNAs. Within the nucleus, they occupy the chromatin fraction. 17% of lncRNAs *vs.* 15% of mRNAs are enriched in the nucleus, whereas 4% *vs.* 26%, respectively, are enriched in the cytoplasm [6]. Many lncRNA-mediated mechanisms of gene regulation have been identified in the cytoplasm [8], [15], [16]. In the last decade or so, thousands of cytoplasmic lncRNAs have been discovered, indicating their importance for multiple cellular activities. In this review we highlight the functions and underlying mechanisms of some important cytoplasmic lncRNAs that are responsible for posttranscriptional regulations such as on mRNA stability and translational control.

## Modulation of mRNA stability

In the cytoplasm, several lncRNAs target mRNA transcripts and modulate mRNA stability. Some lncRNAs such as half-STAU1-binding site RNAs (*1*/*2-sbsRNAs*) and growth arrested DNA-damage inducible gene 7 (*gadd7*) decrease the stability of mRNA, while others such as antisense transcript for β-secretase 1 (*BACE1-AS*) and the terminal differentiation-induced ncRNA (*TINCR*) increase mRNA stability.

### ***1/2-sbsRNAs***

**Figure 1:**
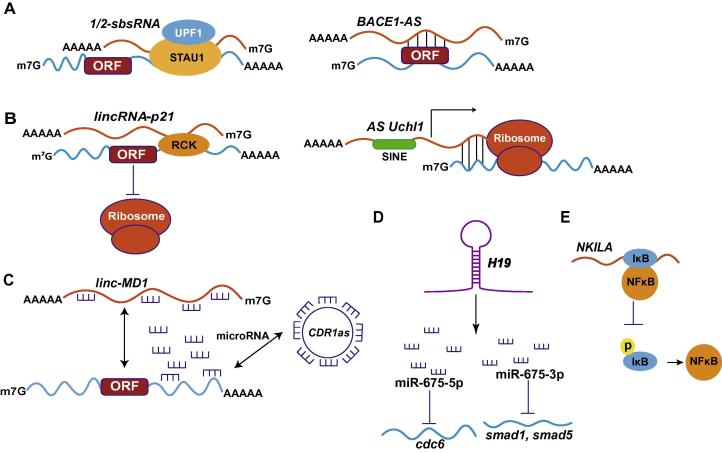
**Known working models of cytoplasmic lncRNA function** **A.** lncRNAs modify mRNA stability, with *1/2-sbsRNA* as an example of decreasing the stability of mRNA and *BACE1-AS* as an example of increasing the stability of mRNA. **B.** lncRNAs regulate mRNA translation, with *lincRNA-p21* as an example of inhibiting the translation and *AS Uchl1* as an example of promoting translation. **C.** lncRNAs modulate gene expression by functioning as ceRNAs, with *linc-MD1* as well as *CDR1as* shown as examples. **D.** lncRNAs can give rise to microRNAs, with *H19* shown as an example. **E.** Some lncRNAs affect protein modification, with *NKILA* as one of such kind of cytoplasmic lncRNAs. ORF, open reading frame; 1/2-sbsRNA, half-STAU1-binding site RNA; BACE1-AS, antisense transcript for β-secretase 1; linc-MD1, long non-coding RNA muscle differentiation 1; CDR1as, cerebellar degeneration-related protein 1 antisense transcript; NKILA, NF-κB interacting lncRNA; AS Uchl1, antisense transcript for ubiquitin carboxy terminal hydrolase L1; SINE, short interspersed element; cdc6, cell division cycle 6.

mRNAs can be degraded via staufen 1 (STAU1)-mediated mRNA decay (SMD), when their 3′ untranslated region (3′ UTR) binds to STAU1 [17]. STAU1 is a double-stranded RNA (dsRNA)-binding protein, which binds within 3′ UTR of translationally-active mRNA [14], [18]. STAU1 binds to a complex structure of 19-bp stem with 100 nt apex within the mRNA encoding ADP ribosylation factor 1 (ARF1) [18]. This stem region is conserved in 3′ UTRs of *ARF1* mRNA of mouse and rat [6]. However, such stem structures were not identified in other STAU1 targets [18]. STAU1-binding sites can be formed by imperfect base-pairing between an Alu element in the 3′ UTR of an SMD target and another Alu element in a cytoplasmic, polyadenylated lncRNA [18]. These lncRNAs transactivate the binding of STAU1 to mRNA as only STAU1 could be immunoprecipitated with lncRNAs called *1/2-sbsRNAs*, thus unveiling a pivotal strategy of recruiting proteins to mRNAs and mediating the mRNA decay (Figure1**A**). However, not all mRNAs containing Alu element in their 3′ UTR are targeted for SMD, despite the presence of complementary 1/2-sbsRNAs that target other mRNA for SMD [17]. One of the 378 identified *1/2-sbsRNAs* in humans, *1/2-sbsRNA1* contains a single Alu element that base pairs with the Alu element in the 3′ UTR of plasminogen activator inhibitor type 1 (*SERPINE1*) and *FLJ21870*. 1/2-sbsRNA1 is present in the cytoplasm but absent in the nucleus of HeLa cells [18] and only STAU1 can be immunoprecipitated with *1/2-sbsRNA1*. Two isoforms of 1/2-sbsRNA1, including *1/2- sbsRNA1*(*S*) (short form) and *1/2-sbsRNA1*(*L*) (long form), have been reported. Both isoforms contain the Alu element and 3’ UTR with poly (A) tail, although they differ at the 5′ end. Knocking down *1/2-sbsRNA1*(*S*) increased the level of *SERPINE1* and *FLJ21870* mRNAs by 2–4.5-folds above normal. Other *1/2-sbsRNA* members such as 1/2-sbsRNA2, 1/2-sbsRNA3, and 1/2-sbsRNA4 are largely cytoplasmic and polyadenylated as well, containing a single Alu element. Knocking down these 1/2-sbsRNAs led to upregulation of their mRNA targets [17]. Functional studies showed that *1/2-sbsRNA1* contributed to the reduced cell migration by targeting *SERPINE1* and RAB11-family-interacing protein 1 (*RAB11FIP1*) mRNAs for SMD as confirmed by scarp injury repair assay [17].

### ***gadd7***

*gadd7* is a 754-nt polyadenylated lncRNA isolated from Chinese hamster ovary (CHO) cells [15], [16]. Expression of *gadd7* is induced by several types of DNA damage and growth arrest signals [19], [20], and *gadd7* plays a pivotal role in regulating G1/S checkpoint post DNA damage. *gadd7* also regulates lipid-induced oxidative and endoplasmic reticulum (ER) stress [21]. lncRNAs are known to bind to and regulate the functions of proteins. One such example is the binding of *gadd7* with TAR DNA-binding protein (TDP-43), and this interaction is strengthened upon UV exposure [22], [23], [24]. TDP-43 is a member of the heterogeneous nuclear ribonucleoprotein (hnRNP) family. HnRNP family members are RNA/DNA binding proteins involved in transcription, splicing, mRNA transport, and mRNA stability [25], [26]. TDP-43 is known to repress the expression of cyclin-dependent kinase 6 (*Cdk6*) mRNA in Hela cells, which is important for G1-phase progression [27], [28]. Nonetheless, *Cdk6* expression is found to be activated by TDP-43 in CHO cells [24]. UV-induced *gadd7* directly interacts with TDP-43, thus leading to the decreased interaction between TDP-43 and *Cdk6* mRNA. This results in *Cdk6* mRNA degradation, and finally inhibition of cell cycle progression [24]. *gadd7* is not highly conserved at the nucleotide level [29], [30]. Since the structure or the functional motif of lncRNAs may be more important, and thus would be more conserved than their nucleotide sequence [9], it remains possible to identify a functional *gadd7* ortholog in humans. This may be important for unveiling the pathogenesis of diseases such as frontotemporal lobar degeneration (FTLD) and amyotrophic lateral sclerosis (ALS), as dominant mutations in TDP-43 are causative of these two important neurodegenerative diseases [24], [31], [32].

### ***BACE1*-*AS***

Expression of the conserved non-coding *BACE1-AS* increases *BACE1* mRNA stability when HEK-SW cells are exposed to cellular stressors like amyloid-β1–42 (Aβ1–42) [33]. *BACE1-AS* renders *BACE1* mRNA stability by masking the binding site of miR-485-5p (Figure1A). *BACE1-AS* and miR-485-5p compete for binding in the sixth exon of *BACE1* mRNA. The sense-antisense RNA duplex between *BACE1* and *BACE1-AS* in the cytoplasm potentially perturb the interaction between miR-485-5p and *BACE1* mRNA, which to some extent, explains the mRNA stabilization by *BACE1-AS* transcript [34].

### ***TINCR***

The *TINCR* gene resides on chromosome 19 in humans and encodes a predominantly cytoplasmic 3.7-kb lncRNA. TINCR regulates human epidermal differentiation by post transcriptional mechanism [35]. Previously found as an uncharacterized lncRNA, *TINCR* is now believed to be the most highly-induced lncRNA during epidermal differentiation [35], [36]. *TINCR* binds to mRNA through a 25-nt ‘TINCR box’ motif, which is robustly enriched in the interacted mRNAs. *TINCR* RNA has a strong affinity for STAU1 protein [17], [35], [37], [38]. *TINCR*–STAU1 complex mediates the stabilization of differentiation-related mRNAs, such as *KRT80* encoding keratin 80 in an ultraviolet protection factor 1/2 (UPF1/2)-independent manner, however the exact mechanism remains obscure [39].

## Modulation of translation

Gene expression control at translational level plays a crucial role in multiple biological systems and provides valuable means for the spatiotemporal management of complex protein dynamics in eukaryotic cells [40], [41], [42]. Some lncRNAs also get involved in such regulation at the translational level, which can either repress (as exemplified for *lincRNA-p21* below) or promote (as exemplified for *AS Uchl1* below) translation.

### ***lincRNA-p21***

The human *lincRNA-p21* is also known as tumor protein p53 pathway corepressor 1 (Trp53cor1). *lincRNA-p21* is ∼3.0 kb in length, and the encoding gene is located ∼15 kb upstream of *p21/cdkn1*a gene [23]. It is more abundant in cytoplasm compared to nucleus, known to co-distribute with ribosomes [43]. As a post-transcriptional modulator, lincRNA-p21 can negatively regulate the translation of *CTNNB1* (β-catenin) and *JUNB* transcripts by imperfectly base pairing at different sites in the coding and untranslated regions (both 5′ and 3′ UTRs) of *CTNNB1* (15 sites) and *JUNB* mRNAs (8 sites). When the level of Hu antigen R (HuR), a ubiquitous RNA binding protein, reduces, *lincRNA-p21* becomes stable and interacts with its target transcripts including *CTNNB1* and *JUNB* mRNAs. The resulting *lincRNA-p21*–mRNA complex can enhance the interaction between mRNAs and the translational repressors RCK as well as Fragile X mental retardation protein (FMRP). Consequently, translation of the target transcripts is repressed through reduced polysome sizes and ribosome drop-off (Figure 1**B**) [43], [44].

### ***AS Uchl1***

A recent study reported the discovery of a spliced nuclear-enriched antisense transcript (*AS Uchl1*) complementary to the mRNA that encodes mouse ubiquitin carboxy terminal hydrolase L1 (Uchl1) [45]. Uchl1 is an enzyme specifically expressed in dopaminergic neurons [24], [46], [47]. The activity of the *AS Uchl1* depends on the presence of a 73-nt overlapping sequence complementary with 5′ end of *Uchl1* mRNA and an embedded inverted SINEB2 repetitive element (Figure1B) [45]. Under normal physiological conditions, *AS Uchl1* is enriched in the nucleus, and upon rapamycin treatment, inhibition of mTORC1 triggers the transport of *AS Uchl1* to the cytoplasm, which then targets the overlapping *Uchl1* mRNA to active polysomes for cap-independent translation. Exact molecular mechanism as to how *AS Uchl1* promotes the translation of *Uchl1* mRNA under stress conditions is still elusive.

## Competing endogenous RNAs

Coding and non-coding RNAs can regulate each other through their ability to compete for miRNA binding. lncRNAs harboring multiple binding sites of identical miRNA are called competing endogenous RNAs (ceRNAs) [48]. ceRNA can sequester miRNAs and therefore protect their target mRNAs from repression [49], [50], [51], [52], [53]. This activity was first discovered in *Arabidopsis thaliana* and later in mammals [53], [54]. Multiple ceRNAs have been identified, and we present some as examples below.

### ***HULC***

Hepatocellular carcinoma (HCC) is one of the most fatal cancers [55]. Recent studies have indicated that a large number of lncRNAs are functionally deregulated in HCC [56], [57], [58], [59]. Among these, highly up-regulated in liver cancer (*HULC*) is a novel mRNA-like ncRNA. It is present in the cytoplasm, spliced, polyadenylated, and resembles the mammalian LTR transposon 1A [60]. As reflected by its name, *HULC* is highly upregulated in HCC, and it is also detected in gastric cancer and colorectal carcinomas that metastasize to the liver [60], [61], [62]. The *HULC* gene resides on chromosome 6p24.3 in humans and is conserved in primates. It is about 1.6 kb in length and contains two exons. Expression of *HULC* gene in Hep3B cells can be up-regulated by the transcription factor cAMP responsive element binding protein (CREB). *HULC* acts as endogenous sponge of miR-372 [63]. *HULC* binding to miR-372 reduces miRNA-mediated translational repression of protein kinase cAMP-activated catalytic subunit beta (PRKACB), one of the target genes of miR-372 [63]. PRKACB can induce phosphorylation of CREB, which in turn stimulates *HULC* expression, thus forming a feedforward loop [63].

### ***linc-MD1***

*linc-MD1* is a muscle-specific lncRNA, which is indispensable for the timing of muscle differentiation and plays an important role in myogenesis [64]. *linc-MD1* acts as a natural decoy for two muscle-specific miRNAs, miR-133 and miR-135 (Figure1**C**) [64]. Expression of mastermind-like-1 (MAML1) is controlled by miR-133, and myocyte-specific enhancer factor 2C (MEF2C) is the target of miR-135 [64]. MAML1 and MEF2C are important myogenic factors required for activation of muscle-specific genes. MEF2C binds to the promoter region of cardiac muscle genes and positively regulates the differentiation of muscle cells [65], [66], while MAML1 acts as a transcription coactivator in some signal transduction pathways (such as Notch signaling) related to muscle differentiation [67]. With the depletion of *linc-MD1*, expression of both MAML1 and MEF2C is repressed, whereas over expression of *linc-MD1* resulted in high levels of MAML1 and MEF2C. These observations argue for a direct competition between *linc-MD1* and mRNAs for miRNA binding [64].

### ***linc-RoR***

The lncRNA regulator of reprogramming (linc-RoR) functions as microRNA (miRNA) sponge against miR-145. Interaction between linc-RoR and miR-145 prevents mRNA of some important transcription factors (TFs) like Oct4, sox2, and Nanog in human embryonic stem cells (hESCs) from miRNA-mediated regulation [68], [69]. The expression of linc-RoR is positively correlated with the undifferentiated state of hESCs [69].

### **CDR1as** and **circSry**

Recently, additional examples of ceRNA were found in circular RNAs (circRNAs), which represent a newly identified large class of lncRNAs [70], [71], [72]. circRNAs can be formed by back-splicing of the 5′ end of an upstream exon with the 3′ end of the same exon or a downstream exon. Although some circRNAs such as EIciRNAs are predominantly localized in the nucleus, circRNAs are generally cytoplasmic. circRNAs appear to be non-coding and lack the association with polysomes [71], [73]. Two cytoplasmic circRNAs have been reported to act as miRNA sponge. The first one is the cerebellar degeneration-related protein 1 antisense transcript (*CDR1as*, also called *ciRS-7*), which is a sponge for miR-7 (Figure1C). *CDR1as* contains 74 miR-7 seed matches, out of which 63 are conserved in mammals [72]. The other one is a testis-specific circRNA encoded by the gene sex-determining region Y (*circSry*), which contains 16 putative binding sites for miR-138 [71], [72], [74]. These two circRNAs may be special cases, and it may not be a general phenomenon for circRNAs to function as miRNA sponges [75].

## Precursor of miRNAs

A genome-wide survey predicted that nearly 100 lncRNAs encode miRNAs [76]. These lncRNAs may not be predominantly cytoplasmic, but they may be processed in the nucleus and cytoplasm to give rise to functional miRNAs.

### ***H19***

*H19* is one of the best known imprinting genes expressed from the maternal allele and required for proper muscle differentiation and muscle regeneration [77], [78], [79]. The H19 gene is present on chromosomes 11 and 7 in humans and mice, respectively [80], [81]. There is no conserved ORF sequence in *H19* RNA between mice and human. Although the H19 gene is imprinted paternally, the *H19* RNA itself does not take part in imprinting mechanism [82]. Studies based on structure prediction suggest that *H19* is a ncRNA, 2.3-kb long, capped, spliced, and polyadenylated [82], [83]. It is reported that H19 lncRNA acts as a molecular sponge for let-7 family of miRNAs in a HEK293 cell line [84]. Depleting *H19* causes accelerated muscle differentiation, which can be recapitulated by let-7 overexpression [84]. In the cytoplasm of undifferentiated multipotent mesenchymal C2C12 cells, H19 interacts with the K homology-type splicing regulatory protein (KSRP). Such binding favors KSRP-mediated destabilization of myogenin transcripts [85].

Besides the aforementioned roles of *H19*, exon 1 of *H19* also gives rise to miR-675-3p and miR-675-5p (Figure1**D**). miR-675-3p targets the gene encoding the anti-differentiation TFs smad1 and smad5, which are crucial components of the bone morphogenetic protein (BMP) pathway [86], whereas miR-675-5p targets the gene encoding DNA replication initiation factor Cdc6 [86]. In this regard, *H19* has a pro-differentiation function in primary myoblasts and regenerating skeletal muscles due to the resulting miR-675-3p and miR-675-5p [86], [87]. *H19* is also found to regulate placenta growth. Insulin like growth factor 2 (*Igf2*), which is also targeted by miR-675-3p, is an important regulator of growth and is upregulated in *H19*-deficient placenta [88]. *H19* is also found to modulate gastric cancer cell proliferation through miR-675, by targeting the gene encoding the tumor suppressor runt domain transcription factor1 (RUNX1). Thus *H19*/miR-675 regulates the expression of *RUNX1* to modulate gastric cancer [89].

### ***linc-MD1* (again)**

We discussed *linc-MD1* as a ceRNA before. However, *linc-MD1* primary transcript also harbors the pri-miR-133b sequence. If cleaved by Drosha in the nucleus, *linc-MD1* can give rise to a miRNA precursor. Recently, HuR protein is described as another component of *linc-MD1* regulatory circuitry [90]. HuR is known to contribute to muscle differentiation [91]. HuR interacts with many coding and non-coding RNAs, indicating its pleiotropic RNA binding activity [92], [93]. HuR binds to and favors *linc-MD1* accumulation at the expense of miR-133 biogenesis. HuR also recruits miR-133 onto *linc-MD1* in the cytoplasm, thereby reinforcing this regulatory circuitry. There is an inverse correlation between levels of HuR and miR-133b. HuR binds the base of the pri-miR-133b stem loop, and physically interferes with microprocessor activity [90]. Further investigations have to be carried out to answer how the processing and function of *linc-MD1* are regulated either as the pri-miR-133b in the nucleus or as sponge for miR-133b when exported to the cytoplasm as an unprocessed transcript.

## Regulation of protein modification

In the recent years, several lncRNAs are identified to modulate modifications of cytoplasmic proteins such as ubiquitination/deubiquitination or phosphorylation/dephosphorylation.

### ***lnc-DC***

Expression of *lnc-DC* is almost exclusive to human conventional dendritic cells (DCs) [94]. *lnc-DC* could help activate STAT3 by binding to it in the cytoplasm, thus promoting the phosphorylation and preventing dephosphorylation of STAT3. Knockdown of *lnc-DC* inhibited the differentiation to the DC lineage as well as the functions of DCs [94].

### ***NKILA***

NF-κB interacting lncRNA (NKILA) binds directly to IκB and blocks IKK-induced IκB phosphorylation, thus inhibiting NF-κB activation (Figure1**E**) [95]. The expression of NKILA is also upregulated by NF-κB. NKILA interacts with the NF-κB/IκB complex, and seems to keep the NF-κB pathway from over-activation and to suppress cancer metastasis [95].

### Another role of ***lincRNA-p21***

*lincRNA-p21* was reported to regulate the ubiquitination of HIF-1α, a transcription factor crucial to hypoxia-induced effects such as Warburg effect [96]. lincRNA-p21 is induced by HIF-1α under hypoxia condition, and binds to both HIF-1α and von Hippel–Lindau tumor suppressor (VHL) protein. Such binding blocks the interaction between VHL and HIF-1α, thus inhibiting VHL-mediated ubiquitination of HIF-1α. This positive feedback loop between HIF-1α and *lincRNA-p21* promotes glycolysis under hypoxia [96].

## Perspectives

lncRNAs are recognized as major regulators in life events such as gene expression, cell differentiation, and tumorigenesis. In this article, we summarized the roles of some lncRNAs in the cytoplasm. lncRNAs can function in the posttranscriptional gene expression such as mRNA stability and translation. Through RNA–protein or RNA–RNA interaction, cytoplasmic lncRNAs could also serve as ceRNAs, miRNA precursors, or modulators of protein phosphorylation.

Recent findings have shown that certain transcripts previously-annotated as lncRNAs in fact can be translated to produce small bioactive peptides [97], [98], [99], [100]. For instance, the conserved micropeptide myoregulin (MLN) was found to be encoded by a skeletal muscle-specific RNA, a previously putative lncRNA [100]. MLN shows structural and functional similarity with SERCA inhibitors, phospholamban and sarcolipin. Interacting directly with SERCA, MLN disrupts the Ca^2+^ uptake into the sarcoplasmic reticulum [100]. Similarly, the endogenous 34-amino acid micropeptide dwarf open reading frame (DWORF) is encoded by another putative muscle-specific lncRNA. DWORF enhances muscle performance by physically interacting with SERCA inhibitors such as phospholamban, sarcolipin, and MLN [100]. These examples demonstrated that some (although maybe limited in numbers) transcripts that are previously annotated as lncRNAs are actually coding, and thus should be considered as mRNAs. Given the vast amount of lncRNAs identified, and many of them are associated with noncoding functions, it is no doubt that more functions and functional working mechanisms are yet to be explored for the large number of cytoplasmic lncRNAs.

Regulation of lncRNA localization is important to coordinate their functions in the nucleus or in the cytoplasm. There should exist machinery either directly or indirectly to transport specific lncRNAs into the cytoplasm, and maybe further to special subcellular locations or complexes. The final localization, concentration, and functions of a specific lncRNA have to be fine tuned by the RNA biogenesis, transportation, degradation, and maybe even modifications. Substantial efforts are required to investigate these aspects.

A single lncRNA can have multiple roles. For example, both *H19* and *linc-MD1* can function as ceRNAs as well as precursors for miRNAs. How these different roles of the same lncRNA are coordinated remains to be addressed. On the other hand, there are undoubtedly more roles and functional mechanisms remain unknown for cytoplasmic lncRNAs. With the extensive investigations of the eukaryotic transcriptome by means of RNA sequencing, most of the lncRNAs including cytoplasmic ones may have already been described. Further studies on these lncRNAs may help to classify them into subclasses based on their biogenesis and functions.

## Competing interests

The authors declare no competing interests.
